# Effect of phospholipid transfer protein on plasma sphingosine-1-phosphate

**DOI:** 10.1016/j.jbc.2024.107837

**Published:** 2024-09-27

**Authors:** Quiana Jones, Jiao Zheng, Zhiqiang Li, Mulin He, Xiang Li, Kezhi Dai, Tilla S. Worgall, Yang Yu, Xian-Cheng Jiang

**Affiliations:** 1 Department of Cell Biology, SUNY Downstate Health Sciences University, Brooklyn, New York, USA; 2 Department of Pathology and Cell Biology, Columbia University Medical Center, New York, New York, USA; 3 School of Laboratory Animal & Shandong Laboratory Animal Center, Shandong First Medical University & Shandong Academy of Medical Sciences, Jinan, China; 4 Molecular and Cellular Cardiology Program, VA New York Harbor Healthcare System, Brooklyn, New York, USA

**Keywords:** phospholipid transfer protein (PLTP), apolipoprotein M (apoM), sphingosine-1-phosphate (S1P), high-density lipoprotein (HDL), albumin

## Abstract

Plasma phospholipid transfer protein (PLTP) is a risk factor for cardiovascular diseases. Sphingosine-1-phosphate (S1P), carried by high-density lipoprotein (HDL), is a potent lipid mediator and is also associated with cardiovascular diseases. We found that germline *Pltp* gene knockout (KO) mice have decreased circulating S1P without influencing apoM, a major S1P carrier on HDL. We then hypothesized that, like apoM, PLTP is another S1P carrier. We established inducible *Pltp-*KO, *Apom-*KO, and *Pltp*/*Apom* double KO mice and measured plasma lipoprotein and S1P levels under different diets. We found that PLTP deficiency, and the double deficiency have a similar effect on HDL reduction. Importantly, we found that all mice have about 50% reduction in plasma S1P levels, compared to WT mice, and PLTP deficiency significantly reduces apoM levels (about 40%), while apoM deficiency has no effect on PLTP activity, indicating that PLTP depletion reduces S1P through HDL reduction. To further evaluate this HDL reduction–mediated effect, we overexpressed PLTP which also caused a reduction of HDL. We found that the overexpression reduces S1P and apoM as well as apoA-I, a major apolipoprotein on HDL. Furthermore, we found that albumin (another reported S1P carrier) deficiency in mice has no effect on plasma S1P. We also found that the influence of PLTP on HDL may not require its direct binding to the particle. In conclusion, PLTP is not a direct S1P carrier. PLTP depletion or overexpression in adulthood dramatically reduces plasma S1P through HDL reduction. ApoM, but not albumin, deficiency reduces plasma S1P levels.

PLTP belongs to a family of lipid transfer/lipopolysaccharide-binding proteins, including cholesteryl ester transfer protein (CETP), lipopolysaccharide-binding protein, and bactericidal/permeability increasing protein (1). It is a monomeric protein of 56 or 80 kDa, depending on glycosylation (2). Besides phospholipids, PLTP efficiently transfers sphingosine-1-phosphate (S1P) (3), diacylglycerol, and α-tocopherol (4). Although CETP can also transfer phospholipids, there is no redundancy in the functions of PLTP and CETP (5). The adipose tissue and liver are two important sites of PLTP expression (2). PLTP also is highly expressed in macrophages (6) and in atherosclerotic lesions (7). Plasma PLTP mediates net transfer of phospholipids from chylomicron and very low–density lipoprotein into high-density lipoprotein (HDL) and also exchanges phospholipids between lipoproteins (8). Additionally, it has been shown that PLTP can act like a putative fusion factor to enlarge HDL particles (9). Interestingly, germline PLTP depletion and overexpression reduce plasma HDL levels (10, 11); however, the former influence the smaller HDLs and the latter influence the larger HDLs (12).

S1P is a potent lipid mediator composed of one long hydrophobic chain and one phosphoric acid group. S1P exerts potent physiological effects through five S1P receptors (S1PR1–5) located on cell membranes (13). S1P is involved in various diseases including atherosclerosis (14) and diabetes (15). S1P circulates in blood and lymph at high (0.1–1 μM) concentrations which are significantly higher than the nM concentrations required to activate S1P receptors (16). S1P in blood is produced primarily by red blood cells (RBC) (17), platelets (18), and endothelial cells (16), and secreted by specific S1P transporter, major facilitator superfamily transporter 2b (18) and S1P transporter spinster homolog 2 (Spns2) (19), respectively. Due to its hydrophobic nature, S1P is poorly water soluble and requires carrier proteins for efficient transport and circulation. According to the literature, most of plasma S1P (45–60%) is carried by apoM and the remainder by albumin (20). However, we also noticed that germline PLTP deficiency also caused a 55% reduction of S1P in the circulation which does not influence apoM levels (3, 21). Based on this observation, we hypothesized that PLTP is one of major S1P carriers, besides apoM and albumin. In the current study, we evaluated the effect of inducible PLTP deficiency, inducible PLTP/germline apoM double deficiency, and PLTP overexpression on S1P in the circulation.

## Results

### ApoM, but not, albumin contributes to S1P concertation in the circulation

**Figure 1:**
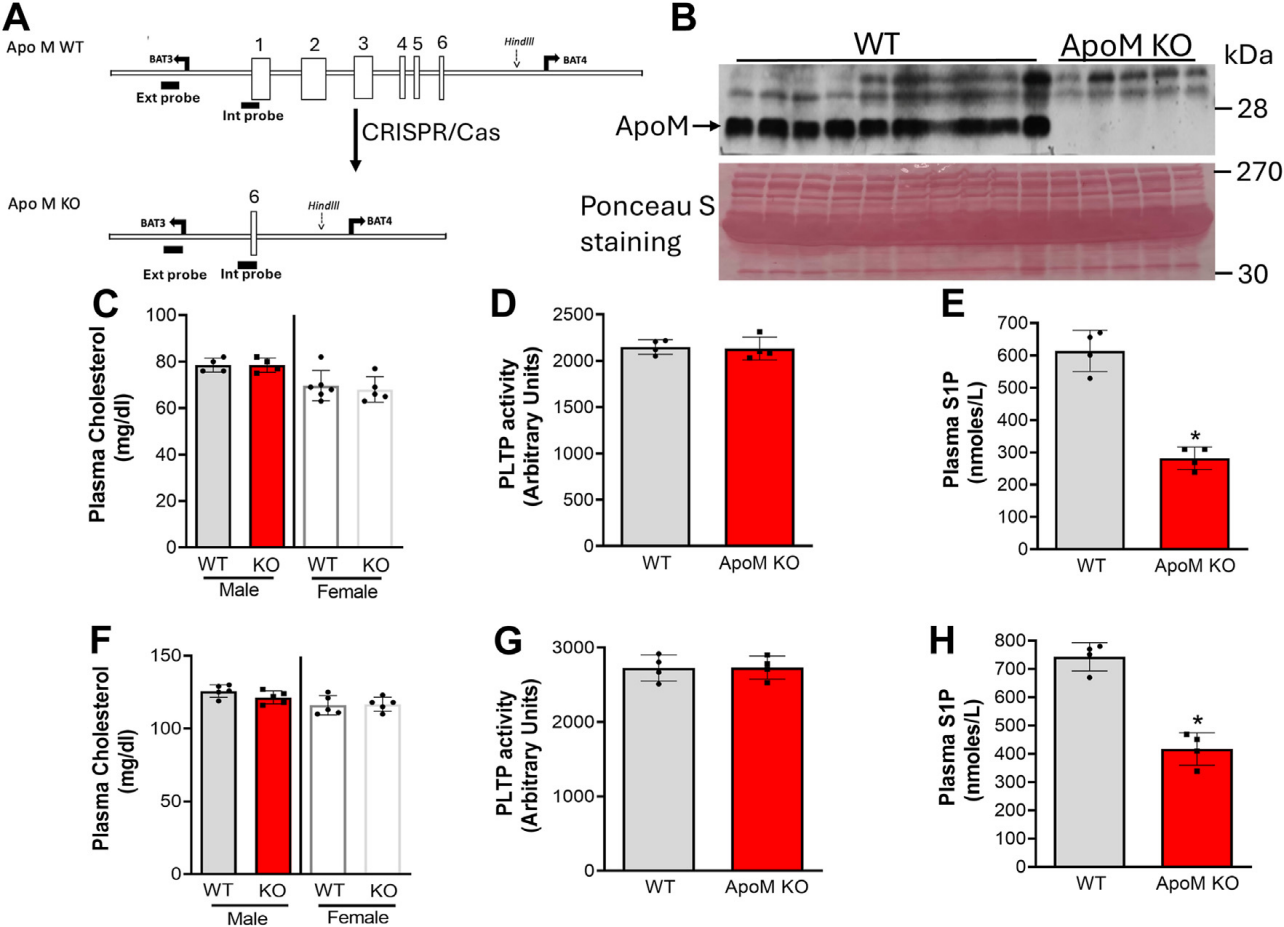
**Lipid and PLTP activity measurements in *Aapom*-KO mice**. *A,* strategy for *Apom*-KO mouse preparation. Three-month-old mice (male and female) on chow diet were used. *B,* plasma (0.2 μl) was separated by 4 to 15% SDS gel electrophoresis and immunoblotted with polyclonal antibodies against apoM. Ponceau S staining was used as a loading control*. C,* plasma cholesterol measurement. *D,* plasma PLTP activity measurement. *E,* plasma S1P measurement. The above mice were then put on the Western diet for 2 weeks and the following were measured. *F,* plasma cholesterol. *G,* PLTP activity. *H,* plasma S1P. Values are the mean ± SD, n = 4 to 5, ∗*p* < 0.01. PLTP, plasma phospholipid transfer protein; S1P, sphingosine-1-phosphate.

It has been reported that apoM is an important S1P transporter in the blood (20). In order to confirm this observation, we used CRISP/Cas9 to prepare *Apom*-KO mice (Fig. 1*A*). We found that no apoM protein is presented in *Apom*-KO mouse plasma (Fig. 1*B*) and apoM deficiency did not change plasma cholesterol levels (Fig. 1*C*), including HDL-cholesterol and non-HDL-cholesterol (Table S1), and PLTP activity (Fig. 1*D*). Importantly, apoM deficiency causes a 55% reduction of plasma S1P levels (Fig. 1*E*) as reported before (20).

Next, we sought to investigate the dietary effect of apoM on S1P. Both WT and *Apom*-KO mice were fed a high-fat high cholesterol (Western) diet for 2 weeks. We then measured plasma lipids as well as PLTP activity. Again, we found that there are no changes in plasma cholesterol (Fig. 1*F*) and PLTP activity (Fig. 1*G*), while plasma S1P were significantly reduced (Fig. 1*H*).

**Figure 2:**
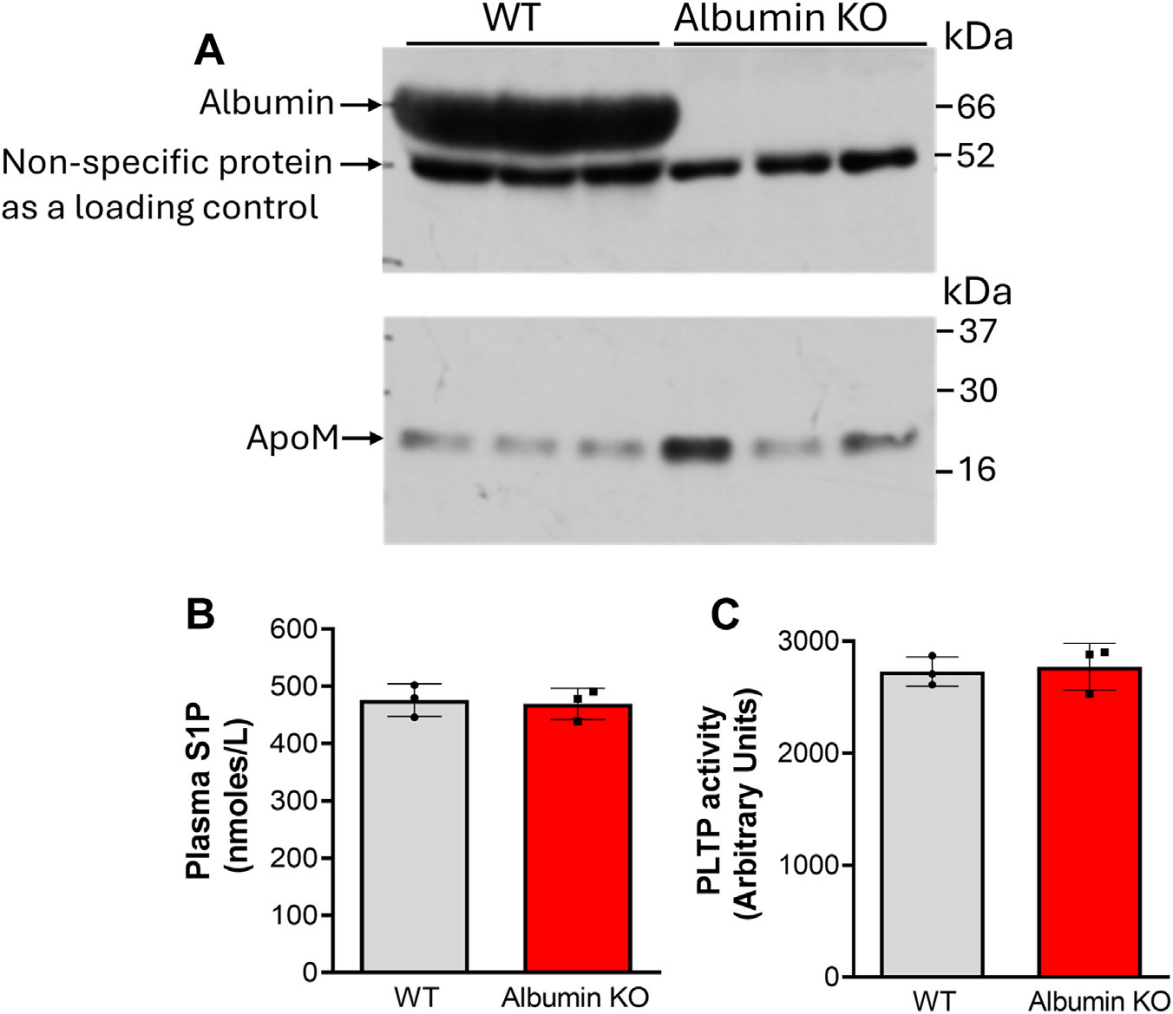
**S1P and apoM measurement in *Alb-KO* mice**. Three-month-old *Alb*-KO- male mice on chow diet were used. *A,* Western blot analyses of plasma albumin and apoM. *B,* plasma S1P measurement. *C,* PLTP activity measurement. Values are the mean ± SD, n = 3. *Alb*-KO, albumin gene knockout mice; PLTP, plasma phospholipid transfer protein; S1P, sphingosine-1-phosphate.

It has also been reported that albumin is another important S1P transporter in the blood (20). We found that *Alb*-KO mice have no albumin in the blood and *Alb-*KO had no significant impact on apoM levels (Fig. 2*A*). To our surprise, albumin deficiency had no effect on plasma S1P levels (Fig. 2*B*). Also, the deficiency did not influence PLTP activity (Fig. 2*C*). Collectively, apoM, but not albumin, makes about 50% contribution to S1P levels in the circulation. There should be other factors that influence the other 50% of S1P in the circulation.

### Inducible PLTP deficiency causes reduction of plasma S1P levels

**Figure 3:**
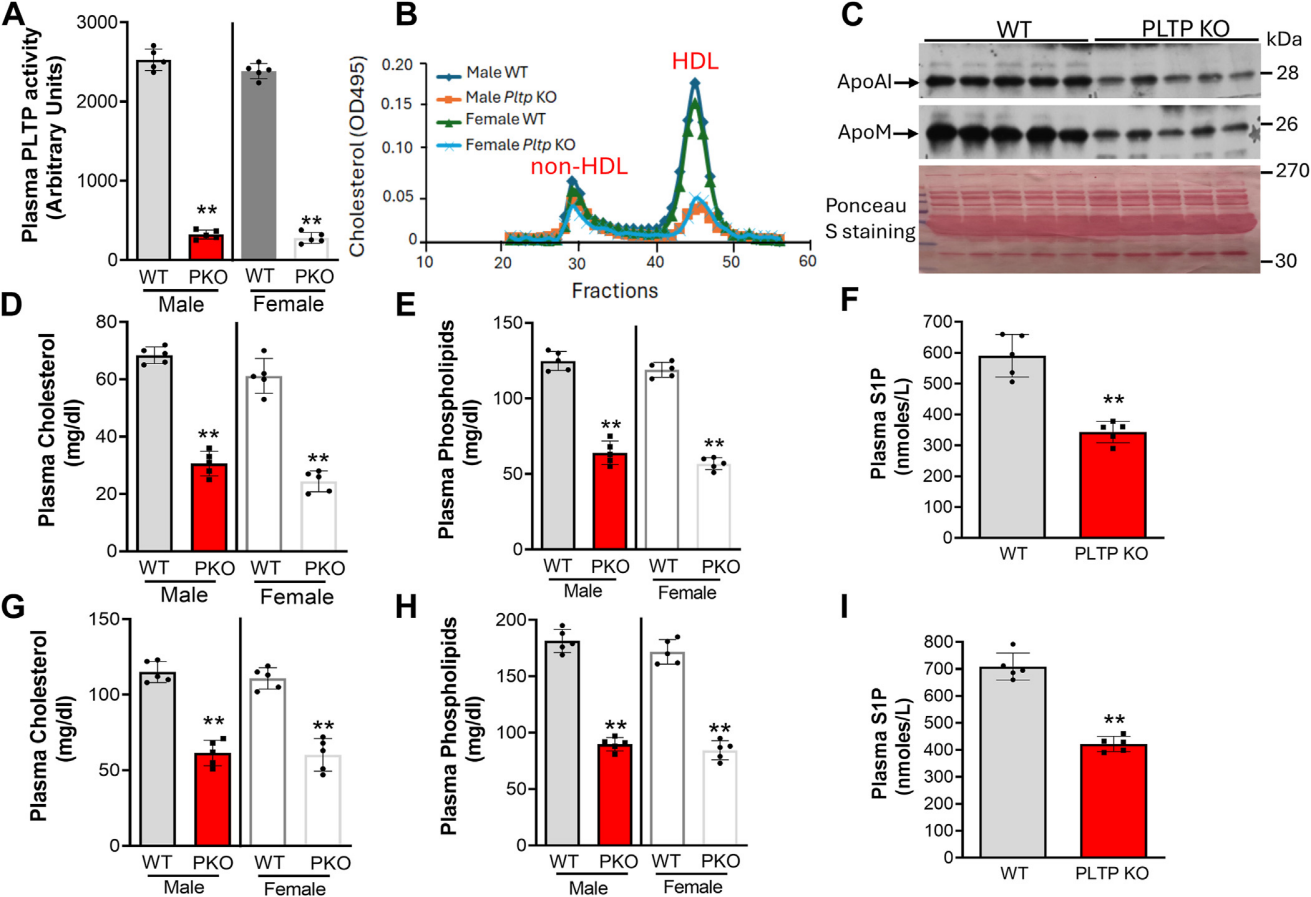
**Lipid and apolipoprotein measurement in inducible *Pltp-*KO mice**. Three-month-old mice (male and female) on chow diet were used. *A,* plasma PLTP activity measurement. *B,* FPLC for evaluating plasma cholesterol distribution. HDL and non-HDL fractions were labeled. *C,* Western blot analyses for plasma apoA-I and apoM (male). The intensity of each band, measured by Image-Pro Plus version 4.5 software, was used for quantification. For apoA-I: 100 ± 7% WT *versus* 55 ± 6% *Pltp*-KO, *p* < 0.01; for apoM: 100 ± 15% WT *versus* 44 ± 5% *Pltp*-KO, *p* < 0.01. *D,* plasma cholesterol measurement. *E,* plasma phospholipid measurement. *F,* plasma S1P measurement (male). Three-month-old mice (male and female) on the Western diet for 2 weeks were used. *G,* plasma cholesterol measurement. *H,* plasma phospholipid measurement. *I,* plasma S1P measurement (male). Values are the mean ± SD, n = 5, ∗∗*p* < 0.01. FPLC, fast protein liquid chromatography; PKO, *Pltp*-KO mice; PLTP, plasma phospholipid transfer protein; S1P, sphingosine-1-phosphate.

Previously, we found that germline *Pltp*-KO mice have a dramatic reduction of plasma S1P compared with WT mice; however, no change of apoM protein levels were observed (3). It is known that depleting a gene in the embryo can lead to compensatory changes in response to the depletion. Thus, we prepared *Pltp*-Flox/UBC-Cre-ER^T2^ mice (22). After tamoxifen treatment, we obtained inducible Pltp-*KO mice (male and female)* with an 85% reduction in plasma PLTP activity (Fig. 3*A*). Fast protein liquid chromatography (FPLC) indicated that the lipid changes were predominantly on HDL fraction (Fig. 3*B*), which was confirmed by Western blot of apoA-I (Fig. 3*C*), a major apolipoprotein on HDL. Unexpectedly, we found that the inducible PLTP deficiency also caused a dramatic reduction of apoM (Fig. 3*C*), an observation which was different from germline PLTP deficiency (3). We directly measured total plasma cholesterol, phospholipid, and HDL-cholesterol. We found that the inducible KO mice have more than 50% reduction in plasma cholesterol and phospholipid levels (Fig. 3, *D* and *E*), mainly on HDL (Table S1). Importantly, plasma S1P levels were significantly reduced (Fig. 3*F*). Moreover, we fed both WT and inducible PLTP KO male mice the Western diet for 2 weeks and then measured plasma lipids. We found that there were increases in plasma lipids, including cholesterol (Fig. 3, *D* and *G*), phospholipid (Fig. 3, *E* and *H*), and S1P (Fig. 3, *F* and *I*) in mice on Western diet, when compared to chow. Again, we found that the deficiency significantly reduces plasma cholesterol, phospholipid, and S1P (Fig. 3, *G*–*I*).

**Figure 4:**
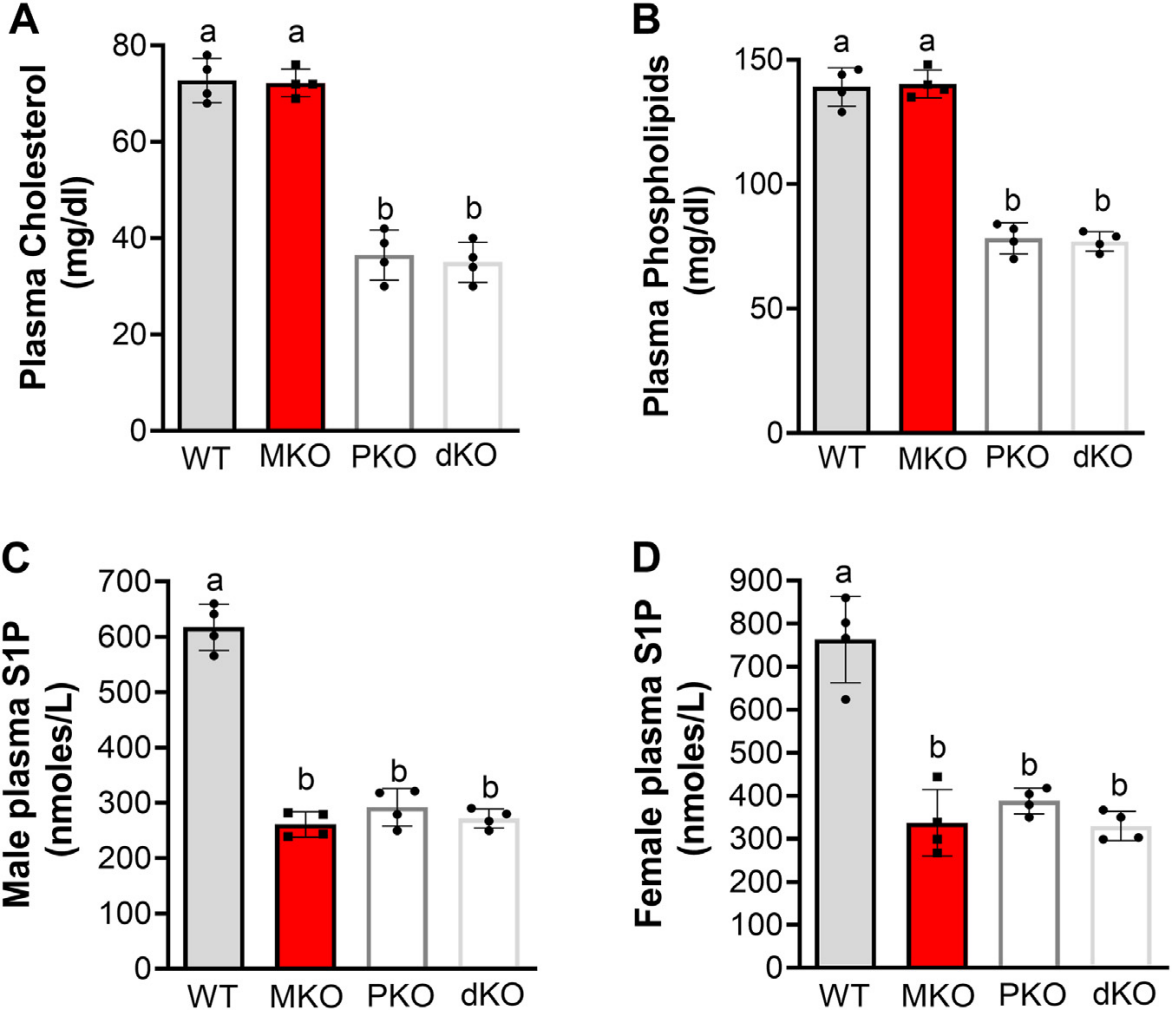
**Plasma lipid measurement in experimental and WT mice**. WT, *Apom*-KO (MKO), inducible *Pltp*-KO (PKO), and inducible *Pltp*-KO/germline *Apom*-KO (dKO) mice on chow diet were used. *A,* plasma cholesterol measurement in male mice. *B,* plasma phospholipid measurement in male mice. *C,* plasma S1P measurement in male mice. *D,* plasma S1P measurement in female mice. Values are the mean ± SD, n = 4. Columns labeled with different lowercase letters are statistically different, *p* < 0.01. dKO, double KO; PLTP, plasma phospholipid transfer protein; S1P, sphingosine-1-phosphate.

In order to further evaluate the effect of apoM and PLTP on plasma S1P, we prepared inducible *Pltp*/*Apom* double KO (dKO) male and female mice. We found that the dKO mice have similar levels of plasma cholesterol and phospholipid as *Pltp*-KO mice (Fig. 4, *A* and *B*). Importantly, the dKO mice have similar levels of plasma S1P as that of *Apom*-KO and *Pltp*-KO mice (Fig. 4, *C* and *D*). In other words, we did not observe an additive or synergistic effect of PLTP deficiency and apoM deficiency, in terms of plasma S1P levels.

### PLTP overexpression causes reduction of plasma S1P levels

**Figure 5:**
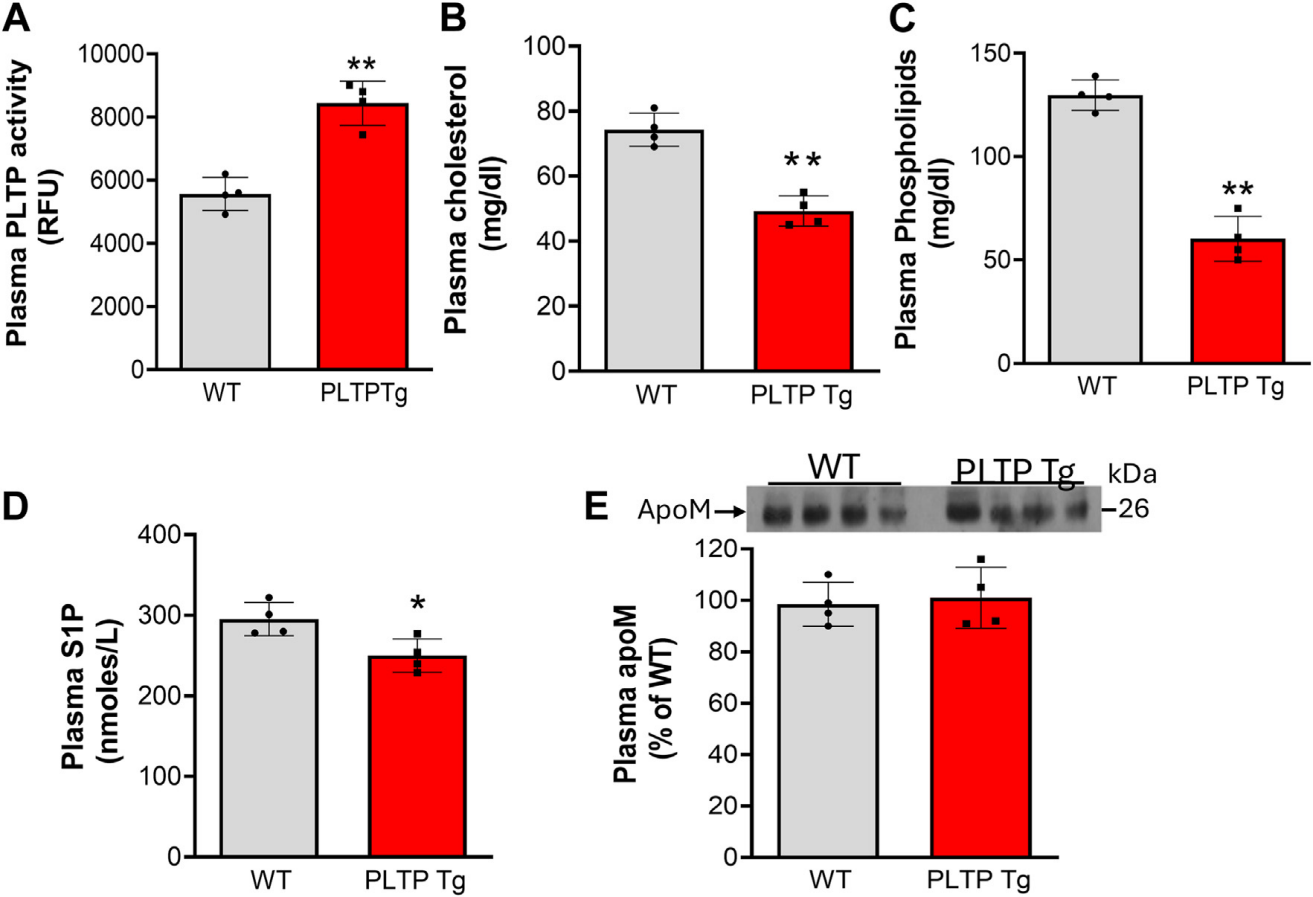
**PLTP activity, lipid, and apoM measurements in germline *Pltp-*transgenic mice**. Three-month-old *Pltp*-Tg and WT male mice on chow diet were used. *A*, PLTP activity measurement. *B,* plasma cholesterol measurement. *C,* plasma phospholipid measurement. *D,* plasma S1P measurement. *E,* Western blot analyses for plasma apoM. The intensity of each band was used for quantification: 100 ± 5% WT *versus* 95 ± 15% *Pltp*-Tg. Values are the mean ± SD, n = 4. ∗*p* < 0.05, ∗∗*p* < 0.01. PLTP, plasma phospholipid transfer protein; S1P, sphingosine-1-phosphate.

**Figure 6:**
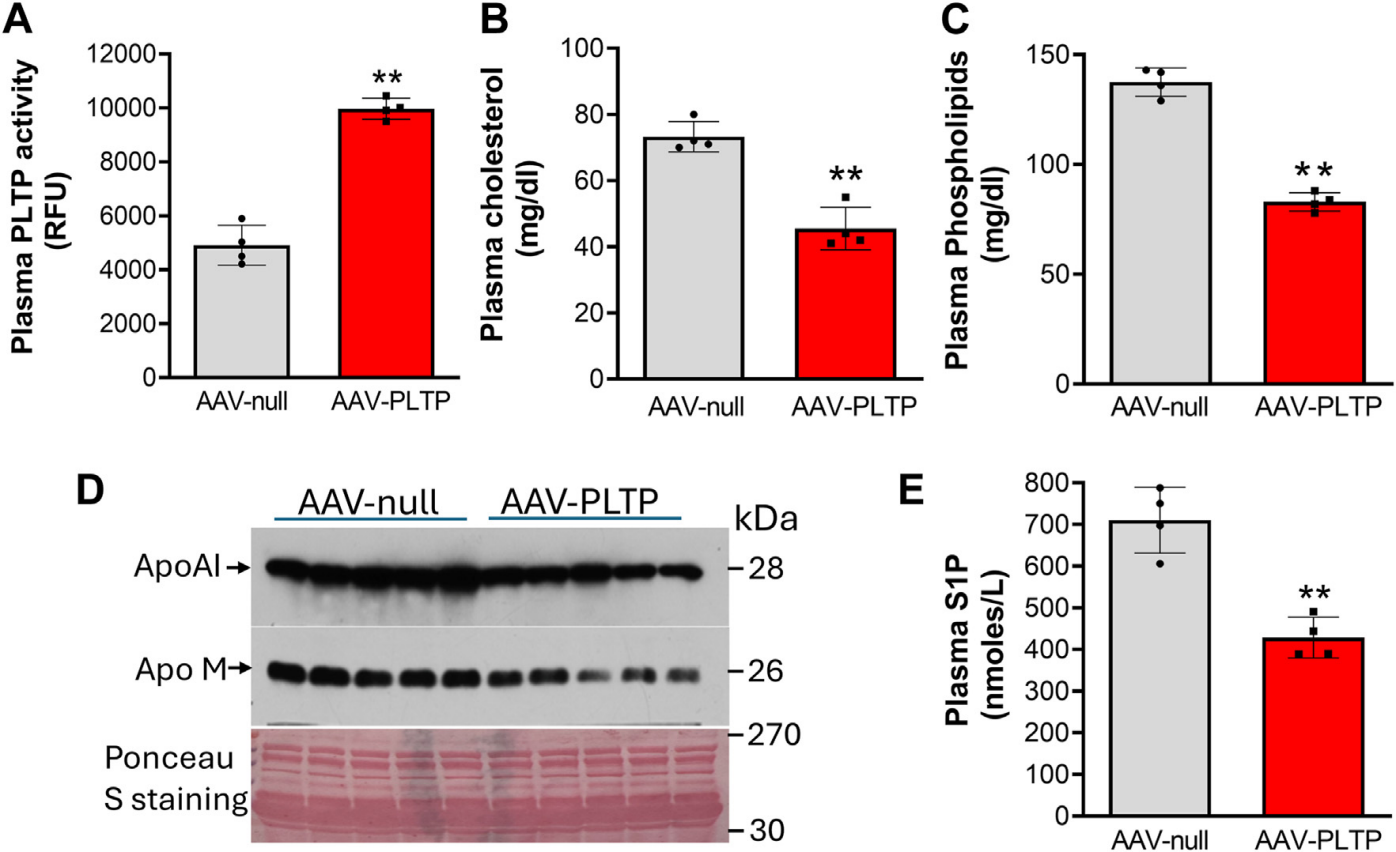
**Lipid and apolipoprotein measurements in AAV-*Pltp* and AAV-null mice**. Two-month-old WT male mice (C57BL/6) were injected with AAV-PLTP and AAV-null (i.v., 2 × 10^11^ per 100 ul). Three weeks later, lipid and apolipoprotein levels were measured. *A,* PLTP activity measurement. *B,* plasma cholesterol measurement. *C,* plasma phospholipid measurement. *D,* Western blot analyses for plasma apoA-I and apoM. The intensity of each band was used for quantification. For apoA-I: 100 ± 10% WT *versus* 65 ± 15% AAV-PLTP, *p* < 0.05; for apoM: 100 ± 8% WT *versus* 52 ± 15% AAV-PLTP, *p* < 0.01. *E,* plasma S1P measurement. Values are the mean ± SD, n = 4 to 5. ∗∗*p* < 0.01. AAV, adenovirus-associated virus; PLTP, plasma phospholipid transfer protein; S1P, sphingosine-1-phosphate.

Since *Pltp*-transgenic (Tg) mice also causes a significant reduction of total cholesterol and HDL-cholesterol (23), we evaluated the effect the transgene on plasma S1P. We confirmed that germline *Pltp*-Tg mice have significantly higher PLTP activity (Fig. 5*A*) and have 40% and 55% less plasma cholesterol and phospholipid levels, respectively, than that of controls (Fig. 5, *B* and *C*). Importantly, we also found that the Tg mice have a 20% reduction of plasma S1P levels (Fig. 5*D*) with no changes in apoM levels (Fig. 5*E*). Since the transgene is expressed in the embryo, we need to know the effect of PLTP overexpression in adulthood. Next, we treated WT mice (C57BL/6) with adenovirus-associated virus (AAV)-PLTP and AAV-null (control) (i.v.) and 3 weeks later, we determined plasma PLTP activity which was significantly increased (Fig. 6*A*). We then measured total cholesterol and phospholipid as well as HDL-cholesterol levels. We found that the PLTP overexpressed mice have about 50% reduction in plasma cholesterol and phospholipid levels (Fig. 6, *B* and *C*), mainly on HDL (Table S1), which is similar to germline *Pltp*-Tg mice (23). Further, we measured apoM levels and found that there is a significant reduction (Fig. 6*D*), which is different from that of germline *Pltp*-Tg mice (Fig. 5*E*). Furthermore, we measured apoA-I levels and found a significant reduction (Fig. 6*D*), indicating a reduction of HDL particle levels. Importantly, PLTP overexpression significantly reduced plasma S1P levels compared to controls (Fig. 6*E*). Collectively, PLTP overexpression–caused S1P reduction could be mediated by its effect on HDL reduction as well.

### Human PLTP plasma distribution

**Figure 7:**
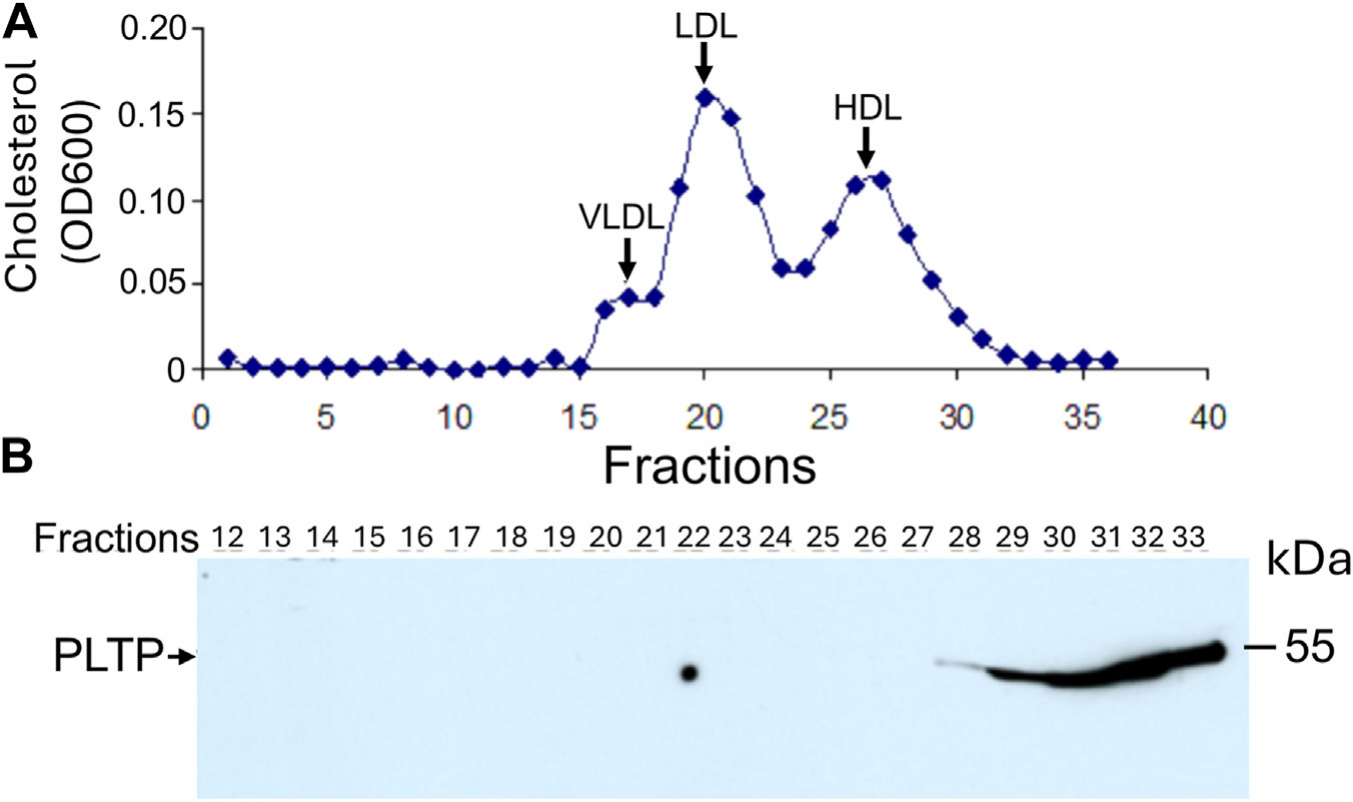
**PLTP distribution in human plasma**. FPLC was performed with human plasma (300 μl). Fractions (1–36) were collected (500 μl/each). *A,* each fraction (100 μl) was used for cholesterol measurement. Based the result, human plasma has three lipoprotein peaks: VLDL (fractions16 and 17), LDL (fractions 19–22) and HDL (fractions 25–30). *B,* we chose fractions 12 to 33, most of them contain cholesterol. Each fraction (30 μl) was used for PLTP Western blot analysis. FPLC, fast protein liquid chromatography; LDL, low-density lipoprotein; PLTP, plasma phospholipid transfer protein; VLDL, very low–density lipoprotein.

It is known that HDL contains PLTP (24), however, the distribution of PLTP in human plasma has not been systematically examined. We performed an FPLC for a fasting human plasma and collected a total of 36 fractions. We measured cholesterol in each fraction and found that there are three cholesterol peaks, that is, very low–density lipoprotein, low-density lipoprotein, and HDL (Fig. 7*A*). We then chose fractions 12 to 33 (containing all three cholesterol peaks) and perform PLTP immunoblot in each of them. As indicated in Figure 7*B*, PLTP is mainly located on cholesterol-free fractions and HDL-cholesterol peak fractions (26 and 27) contain no PLTP at all. Thus, PLTP deficiency– or overexpression-mediated S1P reduction could not be related with its binding capacity with HDL. This is different from apoM which (25) is a lipoprotein-, especially, HDL-bound protein (25).

## Discussion

In this study, we designed experiments which directly compare the effects of decreased and increased PLTP activity on plasma S1P in mice. We found that (1) both PLTP deficiency and overexpression in adult mice cause S1P reduction in the circulation, and both are related with HDL reduction; (2) *Apom-*KO mice, with normal PLTP activity, have significantly less S1P in the circulation; (3) *Alb*-KO mice have no S1P defect; and (4) there is no additive or synergistic effect of *Pltp*-KO and *Apom*-KO on plasma S1P reduction.

It has been reported that 95% of plasma apoM is associated with HDL (26, 27) and apoM carries about 65% S1P in the circulation (28, 29). Interestingly, human apoM was purified and cloned from chylomicron (20), suggesting apoM is also associated with triglyceride metabolism (30). HDL-bound apoM/S1P plays an important role in many biological and pathological processes, such as endothelial cell permeability, inflammation, lipid turnover, and so on (31). Indeed, we confirmed that apoM deficiency causes about 50% reduction of S1P in the plasma under both chow and Western diets (Fig. 1, *E* and *H*). On the other hand, according to the literature, albumin is another major S1P carrier, binding about 30% of S1P in the circulation (28, 29). However, based on our and other researchers’ observations, this may not be true, at least in mice. It has been shown that *Apom*/*Alb* dKO mice have no significant effect on plasma S1P, compared to *Apom*-KO mice (32). We found that *Alb-*KO mice and WT mice have similar levels of plasma S1P (Fig. 2*B*). Collectively, albumin is not a carrier for SIP in mice, a fact which could be true for humans as well. Thus, besides apoM, there should be other factors influencing S1P in the circulation. For example, ApoA4 has been identified as a potential nonlipoprotein S1P carrier (32).

Previously, we reported that germline *Pltp*-KO mice, with no change in apoM, have a dramatic reduction of plasma S1P (60%) and cholesterol (65%, mainly on HDL), compared to WT mice (3). We intended to confirm this observation using the inducible depletion approach. Besides significant reduction in S1P (50%) and cholesterol (mainly on HDL), apoM is also significantly reduced (Fig. 3*C*) which is different from the germline KO mice (3). Moreover, *Apom*/*Pltp* dKO mice have no further reduction for S1P, compared to *Apom-*KO mice, indicating that there is no additive or synergistic effect with the double deficiency on plasma S1P. Given the fact that only about 5% of HDL particles contain apoM (28, 33), and both apoM deficiency and PLTP deficiency result in 50% reduction of S1P in the circulation, respectively (Figs. 1*E* and 3*F*), we suggest that PLTP deficiency associated S1P may be mediated by HDL mass reduction but not apoM reduction.

The HDL reduction–mediated effect on S1P was further confirmed by PLTP overexpression approaches. Germline *Pltp*-Tg mice have a significant reduction in plasma cholesterol, mainly on HDL (23), and S1P but not apoM (Fig. 5). However, AAV-mediated PLTP overexpression in adulthood not only reduced plasma cholesterol (mainly on HDL) and S1P, but also apoM (Fig. 6). Thus, PLTP depletion or overexpression reduces plasma HDL mass, which in turn reduces S1P. Both PLTP deficiency and overexpression result in similar phenotypes, in terms of S1P and other lipids, could mean that PLTP is a component of a large functional protein complex, and the loss or increase PLTP could result in dysfunction of the entire complex. This deserves further investigation.

It is known that common variants at the PLTP locus are strongly associated with human plasma HDL cholesterol levels (34). In mouse models, PLTP deficiency reduces atherosclerosis (35), while its overexpression shows the opposite effect (23). It is still a big puzzle how both depletion and overexpression PLTP in mice lead to a decrease in HDL-cholesterol (35, 36). However, an increase in plasma HDL may not uniformly translate into reduction in heart disease (37). HDL particles are heterogeneous in size and composition (38). Thus, characterizing the different subclasses of HDL mass will be important in clarifying how plasma concentrations lead to cardiovascular disease. In a previous study, we measured size of the HDL in germline *Pltp*-KO, *Pltp*-Tg, and WT mice, and found the order of the size was *Pltp*-KO > WT > *Pltp*-Tg (12). We also measured inflammatory index (derived by dividing net antioxidant activity in the presence of HDL by that observed in the absence of HDL) using HDLs from *Pltp*-KO, *Pltp*-Tg, and WT mice. We found PLTP deficiency had the lowest index, while the overexpression had the highest (12). However, the deletion and overexpression have no influence on macrophage reverse cholesterol transport (39).

S1P has proatherogenic properties. S1P induces inflammation and thrombosis. The S1P gradient facilitates the egress of lymphocytes from lymphoid organs into the circulation and the recruitment of lymphocytes to sites of inflammation (40). S1P activates NF-*κ*B (41), promotes chemotaxis, and stimulates the production of tumor necrosis factor alpha in macrophages and/or monocytes (42). On the other hand, S1P also has antiatherogenic properties. S1P promotes the survival and prevents the apoptosis of endothelial cells mainly through S1PR1 and S1PR3 (43). Endothelium cell–specific S1PR1 deficiency increases mouse atherosclerosis (44). S1PR1 and S1PR2 seems to have opposite effect on atherogenesis, the former is antiatherogenic and the latter proatherogenic (14). Thus, depending on different pathological settings, plasma S1P could be a proatherogenic or antiatherogenic factor. Although, we still do not know the difference between PLTP deficiency-mediated and PLTP overexpression–mediated S1P reduction, in terms of biology and physiology, we know that plasma S1P has both proatherogenic and antiatherogenic properties.

In this study, we also clarified the distribution of PLTP in human plasma, although it has been reported that PLTP is mainly associated with HDL (45, 46). We found the majority of PLTP exist in lipoprotein-free fractions (Fig. 7, *A* and *B*). In other words, the influence of PLTP on HDL may not require its direct binding to the particle. However, this conclusion needs more experimentation to clarify, since HDL fractions still have some overlap with PLTP fractions (Fig. 7).

Although the germline *Pltp-*KO mice have provided extensive information on the role of PLTP in plasma lipid metabolism, the germline deficiency may not be the best technical approach for *in vivo* study. It is known that there are differences between germline deficiency and inducible deficiency on serine palmitoyltransferase (47, 48) and on liver kinase B1 (49, 50). PLTP is another example. We observed the difference between germline *Pltp*-KO (3) and inducible *Pltp*-KO (Fig. 3*C*), between germline *Pltp*-Tg (Fig. 5*E*) and AAV-*Pltp* (Fig. 6*D*), in terms of plasma apoM levels. We think the difference could be caused by PLTP expression time, early life *versus* adulthood. Since both apoM and albumin deficiency also started from early life, we cannot exclude the possibility that the depletion apoM or albumin in adulthood may have different phenotype, in terms of plasma S1P levels. Thus, inducible *Apom*-KO and *Alb* -KO study could be necessary.

In conclusion, PLTP is not a direct S1P carrier, at least in mice, since both PLTP deficiency and overexpression in mice cause S1P reduction in the circulation. However, PLTP activity influences plasma S1P levels through its effect on HDL reduction, and this effect is independent from apoM and albumin levels. Optimum PLTP activity is required for maintaining optimum HDL levels, which influences S1P in the circulation.

## Experimental procedures

### Mice

We crossed *Pltp*-Flox mice (51) with ubiquitin C-Cre-estrogen receptor T2 (UBC-Cre-ER^T2^) transgenic mice and generated *Pltp*-Flox/UBC-Cre-ER^T2^ mice. Tamoxifen (80 μg/g) was intraperitoneally injected into male and female *Pltp*-Flox/UBC-Cre-ER^T2^ mice (12–16 weeks old) to induce the estrogen receptor, which subsequently induced the UBC promoter–mediated Cre recombinase expression. Tamoxifen injected *Pltp*-Flox mice were used as control. The genetic background of the mice used in this study was C57BL/6. The mice were fed a normal chow (Research Diets, Inc). We prepared *Apom*-KO mice through Columbia University Transgenic Animal Facility on the basis of fee-for-service. CRISPR/Cas9 approach was used to target the *Apom* gene, depletion of exon 1 (partial)-6 with CRISPR guides (Fig. 1*A*). Gene-edited founders were generated. Two males were used to cross with WT female mice and both transmitted the disrupted *Apom* allele through the germline. The resulting heterozygous mice were crossed, and the targeted allele was segregated in a Mendelian fashion. The designed target event was confirmed by *Apom* DNA sequencing in both KO mouse lines, in fact, they are identical. Plasma apoM was measured by Western blot and it was completely depleted (Fig. 1*B*). *Apom* and *Pltp* dKO mice were prepared by crossing *Apom-*KO with *Pltp*-Flox/UBC-Cre-ER^T2^ mice, and then treating with tamoxifen.

*Pltp*-Tg mice were prepared by injection (i.v.) of AAV-PLTP (2 × 10^11^ vector genomes in 100 μl). AAV-null was used for controls. Four weeks later, we prepare plasma from the mice to do the analysis. Germline human *Pltp-*Tg mice were a gift from Dr de Crom R (Department of Cell Biology & Genetics, Erasmus Medical Center). *Alb-*KO mice were purchased from Jackson Lab. All animal experiments were conducted under the approval of the SUNY Downstate Medical Center IACUC.

### Lipid and lipoprotein measurements

FPLC was conducted using a 300 μl aliquot of pooled plasma. Samples were loaded onto a Superose 6 increase column and eluted with FPLC buffer (1 M Tris–HCL, pH 7.4 and 0.4 g/l sodium azide) at a constant flow rate of 0.35 ml/min. An aliquot of 100 μl from each fraction was used to measure cholesterol using a kit (Wako Pure Chemical Industries Ltd). An aliquot of 30 μl from each fraction was used to human PLTP immunoblot with a polyclonal PLTP antibody (a gift from Dr John Albers). Plasma total cholesterol and phospholipids were assayed by two kits (Wako Pure Chemical Industries Ltd). HDL-cholesterol was measured according to a published protocol (52): non-HDL was precipitated with sodium phosphotungstate-magnesium (52) and cholesterol in the supernatant was measured as above. Plasma apoM and apoA-I levels were determined as follows. Plasma (0.2 μl) was separated by 4 to 15% SDS gel electrophoresis and immunoblotted with polyclonal antibodies against apoM (Proteintech, Catalog #:12817-1-AP) and apoA-I (Proteintech, Catalog #:14427-1-AP). Ponceau S staining was used as a loading control. Blots were developed by a chemiluminescence detection system (SuperSignal West detection kit, Pierce). The intensity of each band, measured by Image-Pro Plus version 4.5 software (Media Cybernetics Inc; https://image-pro-plus.software.informer.com/4.5), was used for quantification.

### PLTP activity assay

Plasma from tamoxifen-treated PLTP-Flox mice (control) and PLTP-Flox/UBC-Cre-ER^T2^ mice (experimental), male and female, was used to measure PLTP activity as previously reported (12). Donor (2 μl, phospholipid vesicle) and acceptor (50 μl) were combined with 3 μl mouse plasma and 45 μl 10 mM tris, 150 mM NaCl, 1 mM EDTA, pH 7.4. The mixture was then incubated at 37 °C for 20 min. Read fluorescence intensity on spectrometer at excitation wavelength of 465 nm and emission wavelength of 535 nm.

### Plasma S1P measurement

Plasma S1P levels were measure by LC/MS/MS analysis as described previously (53, 54).

### Quantification and statistical analysis

Statistical analysis was carried out using GraphPad Prism version 8.0.2. (https://www.graphpad.com/updates/prism-802-release-notes) Each *in vitro* experiment was independently performed with duplicate or triplicate to ensure reproducibility. Data are shown as mean ± SD. Unpaired two-tailed Student’s *t* test or Mann–Whitney *U* test were performed for two group analyses. Multiple-group comparisons were tested by Kruskal–Wallis followed by Dunn post hoc multiple comparisons tests or Mann–Whitney corrected for multiple tests. *p* values of 0.05 or less were considered to be statistically significant.

## Data availability

All data is contained with the article.

## Supporting information

This article contains supporting information.

## Conflict of interest

The authors declare that they have no conflicts of interest with the contents of this article.
