# Supplementary material for: Effect of phospholipid transfer protein on plasma sphingosine-1-phosphate
Source: J Biol Chem. 2024 Sep 27;300(11):107837. doi: 10.1016/j.jbc.2024.107837 (PMC11532956; doi:10.1016/j.jbc.2024.107837)
Supplement: Supporting information [file mmc1.docx]

**Supporting Information**

Supplement Table 1 Measurement of total cholesterol(C), HDL-C, and non-HDL-C (mg/dl).

----------------------------------------------------------

Mice Total Cholesterol HDL-C Non-HDL-C

----------------------------------------------------------

WT 87+5 59+6 28+4

ApoM KO 82+3 58+5 24+2

Control 89+6 61+7 28+4

iPLTP KO 54+3** 30+3** 24+5

AAV-Null 85+8 63+9 22+2

AAV-hPLTP 51+2** 28+6** 23+3

---------------------------------------------------------- Male mice were used. Non-HDL was precipitated from plasma by phosphotunstic acid and magnesium chloride as described in “Materials and Methods”. Cholesterol in the supernatant was measured as HDL-C. Total cholesterol – HDL-C = non-HDL-C. AAV, adenovirus associated virus; hPLTP, human PLTP; iPLTP KO, inducible PLTP knockout.**P<0.01.
